# The Continued Use of Mobile Health Apps: Insights From a Longitudinal Study

**DOI:** 10.2196/12983

**Published:** 2019-08-29

**Authors:** Isaac Vaghefi, Bengisu Tulu

**Affiliations:** 1 Seidenberg School of Computer Science and Information Systems Pace University New York, NY United States; 2 Foisie Business School Worcester Polytechnic Institute Worcester, MA United States

**Keywords:** mobile health, mHealth, digital health, attrition, law of attrition, continued use, use decisions, goal persistence, IT assessment, smartphone, mobile app

## Abstract

**Background:**

Mobile health (mHealth) apps that support individuals pursuing health and wellness goals, such as weight management, stress management, smoking cessation, and self-management of chronic conditions have been on the rise. Despite their potential benefits, the use of these tools has been limited, as most users stop using them just after a few times of use. Under this circumstance, achieving the positive outcomes of mHealth apps is less likely.

**Objective:**

The objective of this study was to understand continued use of mHealth apps and individuals’ decisions related to this behavior.

**Methods:**

We conducted a qualitative longitudinal study on continued use of mHealth apps. We collected data through 34 pre- and postuse interviews and 193 diaries from 17 participants over two weeks.

**Results:**

We identified 2 dimensions that help explain continued use decisions of users of mHealth apps: users’ assessment of mHealth app and its capabilities (user experience) and their persistence at their health goals (intent). We present the key factors that influence users’ assessment of an mHealth app (interface design, navigation, notifications, data collection methods and tools, goal management, depth of knowledge, system rules, actionable recommendations, and user system fit) and relate these factors to previous literature on behavior change technology design. Using these 2 dimensions, we developed a framework that illustrated 4 decisions users might make after initial interaction with mHealth apps (to abandon use, limit use, switch app, and continue use). We put forth propositions to be explored in future research on mHealth app use.

**Conclusions:**

This study provides insight into the factors that shape users’ decisions to continue using mHealth apps, as well as other likely decision scenarios after the initial use experience. The findings contribute to extant knowledge of mHealth use and provide important implications for design of mHealth apps to increase long-term engagement of the users.

## Introduction

The use of smartphones to deliver health care services has been consistently on the rise for over a decade [1]. Accordingly, this topic has been attracting the attention of researchers and practitioners [2,3]. Given the pervasive and ubiquitous nature of smartphones, their powerful communication and interactive features, and access to internet, which brings an unbounded amount of health information, it is not surprising that mobile health (mHealth) apps have been a topic of investigation in health care. Using smartphones and various apps for transmitting electronic medical records [2], diagnosing and monitoring patients remotely [4], and implementing interventions that help people succeed in areas such as weight management, stress management, smoking cessation, and dealing with chronic conditions [5-8] are examples of mHealth use. Recent surveys show that the market for these apps is rapidly evolving, bringing thousands of apps, aimed at various health purposes, to individuals at a minimal cost [9]. Despite the promise of mHealth apps, the use of mHealth tools has been limited [10], with reports suggesting that most individuals stop using them just before the fifth interaction, and a quarter of mHealth apps are used only once after installation [11,12]. Although promising in its value, it is less likely that the intended benefits of mHealth app use, such as improved access and quality of care, are going to be realized through such short-lived uses of the apps.

Studies that looked at individuals’ adoption and use of mHealth have shown the importance of factors, such as users’ motivation, existing health conditions, individual differences [7,13,14], and individuals’ perceptions about usefulness and ease of use of mHealth [15,16]. Other studies highlighted the importance of design and persuasive nature of these apps and provided frameworks to guide theory-based design and development approaches [17,18]. Although these findings provide valuable insights on users’ decisions to start using a technology, the question remains as to what guarantees the continued use of mHealth apps. This is a critical question, as research on the use of mHealth beyond the adoption phase is sparse [9] and anecdotal evidence has yielded inconsistent findings showing very minimal or no improvements in individuals’ health because of adoption [6,19-21]. It is also a timely question, given the findings in information system (IS) research indicating that use of a system by itself will not be sufficient to provide the expected benefits [22,23]; an information technology (IT) tool should be used more than just a few times [24,25], or it should be used habitually [26], to deliver the expected positive outcomes, such as successful health behavior change. Current understanding of continued use of mHealth apps is limited and there is no study that directly focuses on this important issue.

As such, we conducted a qualitative, longitudinal, and exploratory study on continued use of health and wellness apps, a set of apps that are not disease-specific and aim to promote general wellness. The analysis revealed 2 important dimensions related to users’ assessment of an mHealth app and its capabilities (user experience) and the users’ persistence at achieving their health goals (intent). On the basis of these 2 dimensions, we proposed a 2×2 matrix to depict 4 type of users’ decisions after adopting mHealth, which are to *abandon, limit, switch app,* and *continue use*. The results contribute to health informatics literature by providing a new perspective about how mHealth app use can be continued and the underlying key factors that could facilitate users’ long-term engagement. For health care providers and mHealth app developers, these findings can be used to shape guidelines for better app design, whereas for users, they can ensure continued use decisions versus other possible choices.

The issue of adoption and use of technology has long been pursued by IS scholars. Earlier studies provided an overview of the basic predictors of adoption (see Venkatesh et al [27] for a review), mainly by adopting a cognitive perception-intention-use view to identify factors and antecedents that lead to adoption [28]. For instance, 2 prominent models, Technology Acceptance Model [29] and Unified Theory of Acceptance and Use of Technology [27], have illustrated the key role of several factors, including perceptions about a system’s usefulness and ease of use, attitude [30], motivation [31], performance expectancy, effort expectancy, social influence, and facilitating conditions [27,32] on successful adoption of new technologies. Still, this literature argues that for a technology implementation to be considered successful, and for users to gain significant advantage, it is important that users continue to use a technology beyond the initial adoption stage [33]. Although the same models that were used to explain adoption can be helpful in describing users’ postadoption behaviors [34], the accumulation of research knowledge shows that continued use of technology can be significantly different from initial adoption [35]. Hence, novel approaches were proposed to study the continued use of technology. For example, Bhattacherjee [35] proposed the expectation-confirmation model of continued use and explained that when expectations are positively confirmed via use experience, they can influence perceived usefulness and satisfaction, which increase continued use intentions. Although these studies provide valuable insights, the findings are intentionally abstract and general, so that they are applicable to a wide range of technologies.

mHealth technologies are designed to motivate and persuade behavior change to help users achieve their health and wellness goals. A number of studies have reported on the importance of design in persuasive technologies [36-38] and provided guidelines to improve the effectiveness of such solutions and their adoption [17]. Yet, to our knowledge, there is no study that focuses on the continued use of mHealth apps and the factors underlying this behavior. Our goal is to provide insights on this issue via an exploratory study, which we describe next.

## Methods

### Recruitment

Participants were recruited through an open call in 2 universities (1 public and 1 private) located in the Northeastern United States. We used purposive sampling [32] to select participants that satisfied the inclusion criteria: (1) own a smartphone, (2) be willing to use an mHealth app for at least 14 days, (3) not have had a past history of chronic diseases to ensure that the motivation of participants is not significantly different from typical, healthy mHealth users, and (4) have a specific health goal that can be reached using mHealth apps. We decided to focus on health and wellness mHealth apps because they represent the largest and most-commonly-used category of mHealth apps [3]. All interested individuals were screened over the phone or face-to-face to ensure their compliance with the inclusion criteria. We received approval from our Institutional Review Boards for the study approach described.

### Data Collection

We used semistructured, face to face interviews and daily use diaries to collect data. The interview questions were developed based on a review of existing literature and further refined via discussions with 3 academic experts in health informatics.

During the first round of interviews (preuse interviews), participants answered questions about their approach to health and wellness, motivation to follow a healthy lifestyle, and level of confidence in making lifestyle changes to improve health. At the end of the preuse interviews, we asked participants to (1) identify a health and/or wellness goal toward which they wanted to work during the upcoming 14 days, (2) select a free mHealth app to use and download on their phones, and (3) describe how they are planning to use the new mHealth app.

Given that most users tend to withdraw from mHealth apps before the end of first week [39], we framed the longitudinal study for 14 consecutive days. Longitudinal studies vary in size and complexity, but the continuous monitoring of factors is common among all such studies [40]. This timeframe allowed participants to become familiar with the features of the app and decide whether they intended to continue using the app or abandon it. During the use period, we allowed participants to pick a new app to try if their original choice was not effective in helping them achieve their goals. Every evening, participants received an email with a link to the daily diary survey. The daily use diary included a single question, please describe your interactions with the app today? (eg, How many times and how long you used it? What features did you utilize? Any likes, dislikes?), to capture continuous data about app use. The diary data (see Multimedia Appendix 1 for a response example) allowed us to gather in-depth firsthand accounts of app use from the participants and reduce the likely effect of recall bias.

After the 14-day use period was over, participants were invited for the second round of interviews (postuse interviews). The participants were asked to describe their experience with the app, reflect back on their goals and motivation, assess if the app helped them achieve their goals, and discuss the reasons behind their positive or negative decisions to continue or withdraw use. Finally, the participants were asked to provide design suggestions for app developers that would improve their experience with mHealth apps and result in continued use. The pre- and postuse interviews took, on average, 21 min (13-47 min). The final dataset, collected from May to August 2017, included a total of 34 interviews and 193 daily usage diaries (some participants did not complete between 1 and 3 days of diary keeping). The participants received gift cards at the end of the closing interview.

### Sample Characteristics

Participants were aged between 18 and 51 years, 70% (12/17) females, and 70% (12/17) iPhone users. Among 17 participants, 10 continued using the apps they picked during the initial interviews. However, 7 participants decided to try a new mHealth app because their original choices did not satisfy their needs. This is illustrated in Table 1 along with the participants’ demographic information and areas they decided to focus on during the study.

**Table 1:** Study participant characteristics and goals.

Identification number	Age (years)	Sex	Phone	Selected apps (second choice, if changed)	Area of focus
1	23	F^a^	Android	MyFitnessPal	Diet
2	24	F	iPhone	ARise	Diet/physical activity
3	18	F	Android	Nike+ Training	Physical activity
4	50	M^b^	iPhone	Calorie Counter (Food Diary)	Diet
5	26	M	iPhone	Strong	Physical activity
6	35	F	iPhone	MapMyWalk	Physical activity
7	28	F	iPhone	Sleep Better (TracknShare LITE)	Sleep
8	51	F	iPhone	Weight Watchers	Diet
9	47	F	iPhone	Relax Lite	Mindfulness
10	33	F	iPhone	Aura and Headspace	Mindfulness
11	28	F	iPhone	5 Minute Home Workouts	Physical activity
12	29	F	iPhone	Female Fitness (Fitbit)	Physical activity
13	29	F	iPhone	Plant Nanny and Garmin	Diet (water)/physical activity
14	40	F	Android	Headspace (Aura)	Mindfulness
15	32	F	iPhone	Map my run (HabitBull)	Physical activity/habit building
16	24	M	Android	Samsung Health (Aura)	Physical activity/mindfulness
17	29	M	Android	Charity Miles (ASICS)	Physical activity

^a^Female.

^b^Male.

### Data Analysis

All interviews and individual diaries (over 300 pages) were transcribed and added to QSR’s NVivo application, which was used to code the data and conduct content analysis. To ensure anonymity, each participant was assigned a study identification number (ID). The analysis was performed using the grounded theory approach suggested by Strauss and Corbin (1998). During the open coding phase, we identified a total of 48 codes (eg, app customization, effort needed, reminder/alerts, motivation, activeness, content quality, context access, and continuance/discontinuance) that related to how participants described their use, assessed mHealth apps, and evaluated their willingness to keep using the app. During the axial coding phase, we discussed in multiple rounds how the 48 themes were related or distinct to determine overarching themes; the result was identification of 9 key dimensions that determined continued use of the app. Finally, focusing on the nature of decisions regarding continued use, we proposed a framework (2×2 matrix) based on users’ *assessment of the app* in use and *persistence at health goals.* We provided qualitative appraisal of these dimensions based on information provided and usage patterns described by each respondent, and then mapped the results to the proposed framework.

### Data Exclusion

Among the 19 individuals who were screened, 18 were eligible to participate in this study. A participant was excluded because of existing chronic conditions. Of the 18 eligible participants, 1 dropped out of the study during the 14-day use period and was not included in the analysis (final sample=17).

## Results

### Overview

The literature reports that when testing the influence of behavior change technologies on users’ behavior, characteristics of users should be considered to make sense of the study results [41]. In other words, the user base for an intervention should meet the basic assumptions of the provided technology solution, such as participants’ commitment to making behavioral changes in a specific domain (eg, diet or exercise). In this study, we selected people interested in using health and wellness mHealth apps and making behavioral changes based on a goal they chose, rather than a goal imposed on them. As we studied their interactions with the systems, we observed differences across 2 dimensions. One was their evaluation of the app based on the characteristics outlined earlier, whether enabling or not, and the other was their level of commitment to, and persistence in, achieving the goals they set for themselves. Previous studies focused on these 2 dimensions in isolation. The connection between these 2 dimensions emerged from the exploratory dataset revealing that continued mHealth app use can be influenced by (1) users’ assessment of mHealth apps and (2) users’ persistence at health goals.

### Users’ Assessment of Mobile Health Apps

The first dimension represents the initial user experience with mHealth apps and whether users have a positive or negative assessment of the capabilities of these apps. This dimension resembles previous study findings that highlight the role of satisfaction with IT use as an important step for users to extend the use of technology [35]. Yet, compared with the concept of satisfaction with technology use, which is all-inclusive and abstract and may apply to a wide range of issues related to user and technology interaction, we identified 9 factors and their subcategories that form users’ assessment of mHealth apps through qualitative analysis and multiple iterations of coding. We present these factors, and some exemplary evidence (see Table 2) from the collected data, next.

#### Interface Design

Interface design related comments reflected participants’ preference for *clean and simple* screens and a distaste for cluttered display and overwhelming *advertisements* on the screen. Although users’ understanding of clean and simple may vary, the data revealed that when an interface is crowded with too much text or information, users have difficulty interacting with the app. However, a clean interface helps users navigate the app despite the complex nature of the app. This is relevant to the reduction principle, which suggests reducing complex behavior into simple tasks through the elimination of choices provided, and the liking principle, which suggests designing an interface that is appealing to users in persuasive system design (PSD) framework [17].

#### Navigation

Navigation (how users move through the menus and different features to accomplish their tasks) is another important factor that shapes users’ opinions. The participants articulated their preference for an easy-to-understand *navigation menu and smooth flow* between screens of the app. As PSD framework suggests, reducing complexity is a critical principle to follow while designing successful, persuasive technologies.

The participants also expressed the need for *training* regarding the app’s features and navigation menus (known as wizard) at the beginning of their interaction with the app. When participants reported working with wizards before use, they also reported that it helped them better engage with the app to achieve their goals (eg, participant 10 [P10]; or otherwise, withdraw quickly and look for another app, as they assessed that the app was not in line with their goals; eg, P3).

**Table 2:** Factors influencing decision to continue use.

Factors	Evidence from data
Interface: clean and simple design; appearance of advertisements	“I liked the way the dashboard looked. It was just so clean and so I said ‘Alright well I’ll download that and give it a try!’ and I’ve been really happy with [using] it.” [P12]; “I think the app had a lot of ads, and I know they have to make their money...when I was trying to add something, an ad keeps trying to pop up, it was frustrating.” [P13]
Navigation: navigation menu and flow of pages; training and wizards	“I don’t have to find all these different buttons and how to navigate through it. It’s very simple to use. So, whereas the other app when there’s so many different features...I don’t have time to go through all of them.” [P13]; “Having some sort of quick tutorial orientation...you have to have that...I find it helpful for most apps, so I understand what it does.” [P7]
Notifications: alerts and reminders; control over alerts	“I would expect it to give me text updates or notifications, so I don’t have to go into the app. Because if I have to go into the app [to check my progress] then I would be less likely to check...if it alerts me that would be wonderful.” [P2]; “I mean it actually had a feature that you could set reminders. But...I don’t like any notifications turned on...To me, it is always a distraction...it may work for others.” [P5]; “I think it made me more active, especially because this [referring to a wearable device] has little red lights that pop up. So that kind of forced me hey I haven’t walked for a while or maybe I’ll do a walk around the building!” [P13]
Data collection: data entry convenience; need for extra device	“I mean part of the reason why the step app worked so well was that you literally turn it on it does everything. There isn’t really a lot I need to do to interact with it further.” [P6]; “Yes, that’s the only thing I don’t like right now is that I don’t generally have pockets to carry my phone with me. So, I don’t think it’s accurately reflecting my step count. But if you carry it around it definitely would.” [P12]
Goal management: setting up goals; notifications about progress	“I thought that was one of their big positives. For this app, I think the customizable side of it and being able to track exactly what I wanted is probably its biggest feature and something that I’ve been missing in other apps.” [P7]; “You can click this and then you can go look at your trends over the past several days where here it’s giving you the hourly trend or weekly ones.” [P16]; “You can click this and then you can go look at your trends over the past several days where here it’s giving you the hourly trend or weekly ones.” [P16]; “The app, for instance, sent me emails saying that ‘You have recorded your nutrition for seven days!’ which I found pretty motivating. Kept me going!” [P8]
Depth of knowledge: available content; accuracy of data and content; completeness	“If an [nutrition] app had links to websites that explains how to ferment vegetables, or...links to helpful resources or articles recipes would help me more to get there.” [P4]; “They have a lot of information and you can see kind of like during the night if it spikes when you woke up and it was pretty accurate that way and you could feel like a dream journal and put in you know if you had caffeine late and things like that to kind of track if that affects your sleep.” [P7]; “So, the one app I had initially downloaded I thought had too much locked content, and I felt like I didn’t have enough options. So, I deleted that, and I did download the other app.” [P12]; “But there seem to be no consistent rules. It was overly complicated. I’m like I don’t know how kids would play this. And there was no help document for me to read...and it was vastly inconsistent in terms of the content.” [P6]; “I don’t know if it’s a bug or if it’s supposed to be that way but if you have to pause it doesn't work and it's like you didn’t even do it.” [P14]
System rules: process of the app; clarity of rules and functions	“But there seem to be no consistent rules. It was overly complicated. I’m like I don’t know how kids would play this. And there was no help document for me to read...and it was vastly inconsistent in terms of the content.” [P6]; “I don’t know if it’s a bug or if it’s supposed to be that way but if you have to pause it doesn’t work and it’s like you didn’t even do it.” [P14]
Actionable recommendations: personalized progress analysis; amount of usage time needed	“It did help me become more conscientious about getting some food in me three times a day at least. Becoming more aware of how many calories I was taking in. So, I could meet my goals.” [P4]; “The only thing that [was needed] is to send me related notifications like ‘2000 steps from your goal for the day’.” [P12]
Fit between user and system: match between features and user needs	“I think this app would work for a lot of people. For me what they provide value in, like in their add-ons, does not work. I get [the value] in other places already. So, if I didn't do podcasts, that would be a really nice way to introduce you to walking and running.” [P6]; “From the notification, I knew how I was doing. So, it was nice because I wasn’t doing anything extra to get this information. I didn’t have to go in and really use the app where, with Aura [another app], I had to actively go in and open it up and make the three four minutes for each meditation.” [P17]

#### Notifications

Notifications (*alerts and reminders* triggered by the app) encourage participants to use the app and make their interactions with the app efficient while increasing the likelihood of achieving their goals [17]. Most participants admit that they prefer simple notifications in the forms of smartphone alerts, text messages, or emails that provide a quick overview of their progress toward their health goals.

Although notifications could help motivate more use, the participants prefer some *control* over the frequency and type of notifications they receive. Depending on their preference or motivation to reach their personal goals, some users prefer more frequent notifications that could motivate continued use, whereas others prefer fewer notifications and consider them a distraction. For the latter group of users, notifications could become a deterrent to app use leading to use abandonment.

#### Data Collection

Data collection methods and tools utilized by the mHealth apps are expected to be *convenient* and require low effort, according to the participants. Automatic data collection without the need for users’ frequent input, typically through passive sensing and use of a tracker, can influence the extent of their use and willingness to continue and expand usage of the app.

The analysis showed that efficiency can be achieved when users can quickly interact with a system to perform the intended task. In this study’s context, when an app allows for automatic or quick data entry, it facilitates efficiency in use. This is consistent with the reduction principle in PSD framework [17]. Examples in the data include apps that collect health-related data (eg, step counting) or use guides/templates to speed up the data entry process (eg, default or latest inputs inserted for the user). In addition, all issues related to interface, notification, and navigation directly or indirectly help facilitate and speed up users’ interaction with the app, which all contribute to efficiency.

In cases where manual data entry is unavoidable (eg, tracking diet), users seek features that provide convenient data entry, such as nutrition apps with comprehensive databases that provide nutritional values for a variety of options. Moreover, wearing and *carrying an additional device* all the time acts as a barrier toward full utilization of the system, as some may consider it an additional burden.

#### Goal Management

Goal management is a necessary functionality that enables users to reach their goals [17,18]. The participants expected the apps to allow them to *set goals* and track their performance against their goals. Depending on the context of use, this could translate into setting up daily, weekly, monthly, or longer-term goals that can be used as a reference point for assessing one’s performance. This is related to the self-monitoring principle in PSD framework, which suggests that system features allowing users to keep track of their own performance facilitate progress toward their goals [17]. Examples in the data were defining cups of daily water intake, number of steps to walk, or number of week days to exercise or meditate. Although setting up goals appears to be necessary, many pointed to the need for flexibility in setting up goals, that is, users prefer to have alternative measurement tools and scales to pursue goals. This is related to the tailoring principle of persuasive design, which suggests that tailoring an app to the needs of its users improves its persuasiveness [17]. As an example, a diet management app would need to be able to record weight in pounds or kilograms and use other measurement scales, such as body fat or body mass index (ie, customized goal setting).

In addition, participants expressed that to be able to follow up with their goals, they needed the app to send regular *notifications about their progress*. These notifications, such as text messages, push pop-ups, or emails, help users stay motivated and on track with their goals over time. The analysis revealed that to facilitate continued use, users preferred a progress report (if possible visualized) that hinted at what needed to be done to reach predefined user goals.

#### Depth of Knowledge

Depth of knowledge provided, which refers to the freely *available content* in the app, was a key theme brought up by many of the participants. They distinguished between how much content is freely accessible and the amount of valuable knowledge represented in the app. For instance, a participant expected the meditation app to provide substantial quality content that was freely accessible. The expectation of free quality content appeared to be important when a premium (paid) version of the app was available. The *accuracy* of the information and knowledge represented in the app emerged as another key issue. The analysis showed that when users were not confident about the accuracy of data collected by the app, they would be hesitant to act upon the provided information and move toward the intended goals. Finally, perceived *completeness* of the content provided through the app could determine the extent of users’ interaction with the app. When users perceive the content in the app *sufficient* or *more* than what they believe is needed to reach their personal goals, they tend to use it. This is consistent with the expertise principle discussed in PSD framework [17]. However, if the app does not provide sufficient information regarding achieving goals, or requires a payment to unlock the content, especially if users believe that the amount is unjustified, it blocks the opportunity for continued use because of loss of credibility.

#### System Rules

The clarity of the system rules embedded in the technology, in other words the way the system is designed to work, emerged as a major theme in the analysis. Although the specifics may vary in each app and context, users expect to easily understand the process underlying the design of the app. When the *rules and functions* represented in the app are clear, the user can make an informed decision to commit to the app and continue using it toward achieving personal goals. If users do not understand the process of how a system works based on their initial interactions, they will not be motivated to continued use.

#### Actionable Recommendations

Although the participants used apps with different focuses and features, they stated that to realize the benefits, the apps needed to provide actionable recommendations for improving the current conditions and specify what needed to be done to reach the goals in the intended timeline. This refers to the system’s ability to offer clear next steps that users can follow to reach their goals and is relevant to the tunneling principle in PSD framework, which suggests guiding users during the change process by providing means for action that helps them get closer to their goals [17]. When the app does not summarize user data and provide reports, individuals will have difficulty making sense of their actions, and hence, will not be motivated to continued use. Providing *personalized progress analysis* (a large range of relevant analyses based on the data collected from users) can assist users in tracking their goals and keep them motivated and engaged with the app, leading to continued use.

The *amount of usage time* needed to interact with an mHealth app, for instance, to input data, check progress, or get feedback, to receive accurate progress reports and actionable recommendations toward reaching health goals should be aligned with users’ expectations. On the basis of the goals set by the participants, the time needed for interacting with their apps varied. Our analysis showed that to continue use, it was important that the users perceived the required amount of time needed to work with the app as adequate. When there is inconsistency between the time a user can allocate to the app versus the time required by the app, users tend to withdraw from it after a period of time. As personal mHealth app use is not mandatory, it is important that users be able to spend sufficient time with this technology to reach the intended outcome.

#### Fit Between User and System

Finally, although every app embeds a different set of features and provides various functionalities, there should be a *fit between user and system*, defined in terms of the match between user attributes and app attributes. Our analysis shows that when there is a good match between user attributes (such as preferences, expectations, and personality traits) and app attributes (such as interface, features, content, navigation, and rules), the app’s use will be continued. When the app offers users an opportunity to achieve their health goals, users may extend and continue their use of the app beyond the first few interactions.

### Users’ Persistence at Health Goals

The second dimension related to continued use that emerged from the analysis was the intent of the users. In addition to the factors related to the technology being used, motivation of users played a vital role in continuing to use an mHealth app. Having persistence at intended health goals and being able to pursue them despite the likely challenges appeared to play a key role toward continued use of mHealth apps. Behavior change is difficult to achieve, even with the use of persuasive technologies [17]. For instance, 1 of the participants reported that although she found that the app had powerful features, she did not have enough motivation to continue use beyond the scope of the study. In our sample, although all users committed to a health goal at the beginning of the study (see Table 1), the analysis of the exit interviews revealed that not all demonstrated persistence toward the goal throughout the course of the study (see Multimedia Appendix 2 for examples). The mHealth apps we studied are autogenous technologies that people choose to use to change their own behavior [17]; hence, the use is completely voluntary and self-motivated. Previous research has shown that when there is no mandate to enforce the use of a technology, people have less tendency or positive attitude toward using that technology [42], unless internally motivated to do so [43]. In such a situation—common to mHealth apps—those who have higher persistence toward reaching their goals (stronger intent) appear to have longer continued engagement with the technology.

### Typology of Mobile Health App Use Decisions

The results revealed 2 dimensions that were related to the continued use of mHealth apps. The first dimension considers an overall assessment of user experience and how an mHealth app provides opportunities for reaching health goals through the factors identified in the results section. This subjective assessment made by users can vary from high to low, depending on the extent to which technology is enabling them to achieve the intended goals. For instance, a user may believe an app is a high enabler because it provides notifications, has a simple interface, and allows for automatic data entry techniques, whereas another user may believe the same app is a low enabler because of the insufficient health information the app provides. The second dimension considers the intent of a user by assessing their level of commitment to their health goals. This is usually demonstrated by assessing the extent to which the users exhibit undivided attention and persistent efforts toward achieving goals and could range from low to high.

Considering these 2 dimensions, we identified 4 possible scenarios as an outcome of users’ initial experiences with mHealth apps, which are the decisions to (1) abandon use, (2) limit use, (3) switch app, and (4) continue use.

#### Abandon Use

The decision to abandon quadrant (low assessment and low persistence) represents a situation where users are skeptical about the capability of the mHealth apps they selected, but, at the same time, they do not show persistence at their health goals. In such conditions, we expect a user to abandon the mHealth app before having any meaningful interaction with it. An example is a user who shows willingness to *improve own’s well-being* but withdraws from this goal when faced with an obstacle, for instance, going 1 day without exercising, while simultaneously assessing the app as insufficient for reaching that health goal. As a respondent put it:

I feel like having only one means of communication or accountability is not good for me. I think if I'm serious about it, then I need to go to the meetings and be more engaged. Even though the system holds me accountable for it, it was not enough.

P8

#### Limit Use

The decision to limit use quadrant (high assessment and low persistence) refers to a situation where an enabling mHealth app is available, yet users do not show persistence for pursuing their goals and stop use when they experience any difficulty. An example is a user who reports having a short and intermittent interaction with the selected app, although he or she found the app suitable for reaching the goals. In this situation, we expect users to have a limited use of the app, insufficient for significant improvement toward achieving goals.

#### Switch App

The decision to switch quadrant (low assessment and high persistence) refers to a situation where users show commitment toward their goals but find the mHealth app to be a low enabler because of limited features of the app. In such situations, we expect a user to continue use but substitute that app for a better choice. For instance, a respondent admitted that “it [my use] depends on whether I find the app useful or not, because meditation is something that seems really helpful for me and I’d like the idea, but the [app] implementation isn't working so well…so, I'll go try something else and see.” (10). Although substituting can be distracting, it can still provide an opportunity for reaching goals if a better mHealth app is found and then used in the future.

#### Continued Use

**Figure 1:**
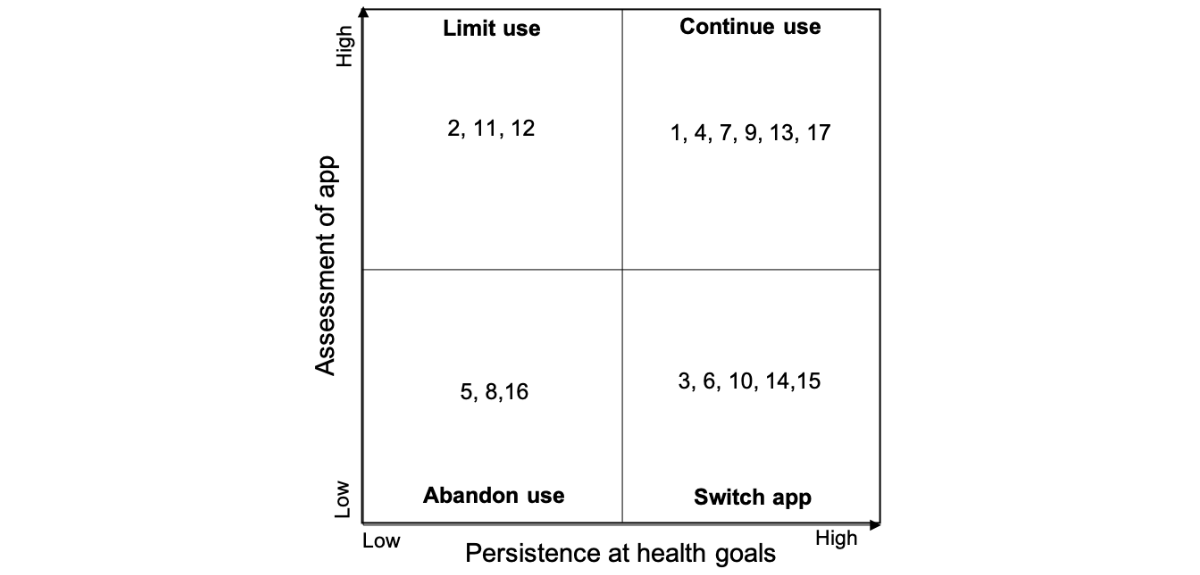
Use decision scenarios regarding mobile health app use.

The decision to continue use is indeed the ideal situation, where the app is enabling and users show persistence toward goals. Under this condition, we expect the users to continue engagement with the app for longer periods, which eventually helps them move toward their intended goals. Figure 1 illustrates our assessment of the participants (the assigned ID), according to the qualitative review of their data.

On the basis of these findings, we developed 4 propositions that describe circumstances associated with the decisions mHealth users make.

*Proposition 1:* When individuals have low assessment of the mHealth app and low persistence at their health goals, they will abandon using the mHealth app.

*Proposition 2:* When individuals have high assessment of the mHealth app and low persistence at their health goals, they will limit their use of the mHealth app.

*Proposition 3:* When individuals have low assessment of the mHealth app and high persistence at their health goals, they will switch to a different mHealth app of their choice.

*Proposition 4:* When individuals have high assessment of the mHealth app and high persistence at their health goals, they will continue to use the mHealth app.

## Discussion

### Principal Findings

Despite the exponential rate at which new mHealth apps are introduced to market, most users stop usage soon after initial use. The aim of this study was to further our understanding of continued use of mHealth apps. Through the analyses of qualitative data collected via interviews and daily use diaries, we identified key factors that influenced users’ decisions regarding continued use after the initial interaction with an app. Furthermore, based on the degree of users’ assessment of the app and their persistence toward their goals, we highlighted 4 decisions: to abandon use, to limit use, to switch app, and to continue use. We put forth propositions that can guide future research that aims to understand behaviors regarding the use of mHealth apps.

### Strengths

mHealth apps will continue to play a pivotal role in providing individual and customized health care services that can be reached anywhere, any time and at relatively low costs [7]; yet, the challenges regarding individuals’ short-term use of these apps impede achieving the intended outcomes and making behavior changes. The exploratory study revealed that following PSD principles [17] can help improve the design of future technologies. In line with *less is more* recommendations in human-computer interaction literature [44], the results highlighted the importance of clean and simple interfaces as the gateway for users to have direct and straightforward interactions with mHealth apps. Clear rules, easy navigation through different parts of a system, and navigation wizards encourage continued use. Automatic data collection and simple data entry methods such as automatic food suggestions improve not only users’ interactions with the app but also their satisfaction with the experience. Ultimately, when there is a good fit between users’ needs and mHealth apps, continued use is likely to occur. Nonetheless, these practical implications should be considered with caution, as it is very challenging to consider all these factors at the same time when designing an mHealth app. Therefore, future research can investigate varying conditions and app-related characteristics that are relevant to each or a subset of factors promoting continued usage, which will provide a more granular view of the identified factors. Overall, these findings echo the calls for user-centered and goal-directed design approaches in the previous research [45]. The findings presented these design principles and concepts as perceived by the users during their decision-making process. Although the study provides important insights to better understand the underlying factors of continued mHealth app use, further research is needed, for instance, using surveys and larger sample, to test the effect of these relationships in various mHealth use contexts.

More importantly, the findings highlighted the importance of focusing on users’ goals and their commitment to these goals. Although motivation is shown to be sufficient for adoption of mHealth [13,46], we found that users who had persistence throughout the usage period were more likely to continue using the app and experience its positive outcomes [33]. As the use of mHealth apps is typically voluntary, their use should be proactively pursued. The study illustrates that persisting at goals while using the right system that fits users’ needs could facilitate continued use, which could pave the way for achieving improved health outcomes. At the same time, we reveal other use scenarios that could result in the absence of goal persistence or lack of fit.

### Limitations and Future Research

We acknowledge that this study has limitations. First, more than half of the participants were female, used an iPhone, and were highly motivated to take care of their health. These characteristics may have influenced the way they interacted with, and made decisions about, the mHealth app. Including larger dataset in a population (eg, balanced male/female; iPhone/Android; and motivated/unmotivated) will help improve the generalizability of the findings. Second, although we used 2 methods to collect longitudinal data, the provided information was self-reported and did not include objective measures. Using system log data in future studies may help provide additional insights on continued use of mHealth apps. Third, we focused on a limited range of mHealth apps (ie, health and wellness) as representative of mHealth apps. Future research is needed to replicate and extend the results to other contexts to have a more inclusive view of the continued use of mHealth. Fourth, participants were recruited in universities (although only 1 was a student) and received compensation for their participation.

In addition, we sent a daily reminder to participants to fill out their use diary, which could have influenced their interaction with the app and encouraged continued use. In the same vein, we acknowledge that other factors could influence continued use of mHealth app. For instance, previous research has shown the importance of privacy regarding adoption and use of mHealth app [47], yet privacy was mentioned only marginally in the dataset. Although such limitations are a common issue in exploratory research [32], further confirmatory studies are needed to validate and generalize the results in broader settings.

Finally, we note that the data focused on continued use as one way to successfully change the health behavior of individuals, yet we did not directly assess the behavior change of users per se. This presents a promising avenue for additional studies, for instance, using longer scope and more comprehensive data collection methods that pay specific attention to the relationship between continued mHealth use and health behavior change to assess how the former behavior instigates the latter.

## Appendix

Multimedia Appendix 1 A diary response example.

Multimedia Appendix 2 Coding structure in qualitative analysis.

## Abbreviations

ID: identification number
IS: information system
IT: information technology
mHealth: mobile health
PSD: persuasive system design
PX: participant X
