# Supplementary material for: The Continued Use of Mobile Health Apps: Insights From a Longitudinal Study
Source: JMIR Mhealth Uhealth. 2019 Aug 29;7(8):e12983. doi: 10.2196/12983 (PMC6740166; doi:10.2196/12983)
Supplement: Multimedia Appendix 1 [file mhealth_v7i8e12983_app1.pdf]

Appendix 1 – A Diary Response Example

**Please type in your name**:<br>[#1]
**Please describe your interactions with the app today. (E.g., How many times and how long you used it? What features did you utilize? Any likes, dislikes?)**<br><br>Today, I logged into the app only twice, but I recorded all of my meals. I have been traveling the past few days and although I have data I prefer to do my app browsing/emails when I am connected to the WIFI, which partially explains why my log-ins and responses have not been as frequent (I have been keeping track of my activity on a notes page in my phone and then submitting my daily interactions into the survey). In addition, since I am with friends it is more difficult to log my meals as I eat them because I am eating with other people and I find it distracting/almost rude to have your phone at a restaurant table when in the company of others. Generally, I eat my meals alone or watching TV/on the computer; I find it to be more of a challenge to integrate the MyFitnessPal app into my eating routine if I am eating out with others or socially.
