# Supplementary material for: The Continued Use of Mobile Health Apps: Insights From a Longitudinal Study
Source: JMIR Mhealth Uhealth. 2019 Aug 29;7(8):e12983. doi: 10.2196/12983 (PMC6740166; doi:10.2196/12983)
Supplement: Multimedia Appendix 2 [file mhealth_v7i8e12983_app2.pdf]

Appendix 2 – Coding Structure in Qualitative Analysis

Sample Responses	Initial Coding	Final Code
• *I don't like overcrowded screens. I like that I can just flick this out of my pocket and the. Numbers are right there. Boom. Huge. You know it's not distracting font you're not going to break down. (#6)*<br>• *But my friend had posted a picture on Instagram or Facebook or something of her Fitbit dashboard right around the time I was doing this, and she was like "oh I just love this" … I liked the way the dashboard looked. It was just so clean. (#12)*<br>• *Less features. There are just too many buttons and I just don't have time for that. I just need steps. And so just a little too much. (#13)*	Clean and simple Design	Interface
• *I think the plant nanny app had a lot of ads. and I know you have to make their money somehow it is free. So maybe you have the option of charging like 99 cents for an ad free version so that it's not crashing. (#13)*<br>• *The first app I picked was full of ads… I liked this one because …. it doesn't spam you with ads, which I guess because they're Nike they can do that. (#3)*	Appearance of advertisements	
• *It was easy to find things I think, they give you good options to explore different features, so you can get an idea about what you could do with it. (#16)*<br>• *I don't have to find all these different buttons and how to navigate through it. it's very simple to use. So, where's the Garmin one when there's so many different features I guess I don't have time to go through all of them and say "oh yeah I'm going to use this. (#13)*	Navigation menu and flow of pages	Navigation
• *The number one thing pretty much across the board is I would like to have more help documents. (# 6)*<br>• *So, having some sort of quick tutorial orientation you have to have that I find helpful for most apps. (#7)*<br>• *I think it was easy to use because in the beginning of downloading the app it kind of gave me a step to what this is .... It explained everything. (#2)*	Training and Wizards	

Sample Responses	Initial Coding	Final Code
• *I would expect it [app] to give me text updates or notifications, so I don't have to go into the app. (#2)*<br>• *At the end of the day at some point you will say oh you reach your goal right. So that was something I liked because I get the notification. (#16)*<br>• *I think definitely necessary are reminders…whether it's an alarm or something else. I remember Fitbit had alarms if you're not moving enough it will go ding ding ding…(#8)*<br><br>• *I mean it actually had a feature that you could set reminders. But ... I don't like any notifications turned on...To me, it is always a distraction. (#5)*	Alerts and reminders<br><br>Control over alerts	Notification
• *Part of the problem that I run into is data. Because I'm running around so much that identifying it [the food] and putting the right food is difficult. (#8)*<br>• *I mean part of the reason why the step one worked so well is that you literally turn it on it does everything. There's not really a lot I could do to interact with it further. (#6)*<br><br>• *Yes, that's the only thing I don't like right now is that I don't generally have pockets to carry my phone with me. (#12)*<br>• *…because I know just my cell phone in general, if I have in my pocket all day it will track your steps maybe not as accurately but at least you have a ballpark. I think it would be nice to have that if you forget your wristband. (#13)*	Data entry convenience<br><br>Need for extra device	Data Collection Methods and Tools
• *There reason I am using this app is because I saw how easy it was to setup. I just had to put in my name, my weight, my height, and it has what I'm looking for so I'm going to check my calories and nutrition and see so it tells me what I'm missing. (#2)*<br>• *I thought that was one of their big positives. For the T&S app I think the customizable side of it and being able to track exactly what I wanted is probably its biggest feature and something that I've been missing in other apps. (#7)*<br><br>• *You can click this and then you can go look at your trends over the past several days where here it's giving you the hourly trend or weekly ones. (#16)*<br>• *This app worked because it focuses on what matters to you "meditation made simple learn in just 10 minutes a day". It also helped me track my progress to stay motivated. (#10)*	Setting up goals<br><br>Notifications about progress	Goal Management

Sample Responses	Initial Coding	Final Code
• *If an [nutrition] app had links to websites that explains how to ferment vegetables, or …links to helpful resources or articles recipes would help me more to get there. (#4)*<br>• *The first one and I liked that it was geared toward women in the beginning. And then I didn't like all the locked content. You have two options under each like exercise that were free and everything else is locked. (#12)*<br>• *There is no record of what I have done without paying more money. There's a $4 upgrade you could do if you wanted to actually record all of your stats. So, in each workout there are usually about five exercises out and then at the end of the exercise it would ask you to record either how long you did it for/how many reps you did depending on the type of exercise. And then at the end of the workout if you want to save the data pay for $4…so I stopped entering the data (-TI). So, I kind of lost the motivation to keep doing it. (#11)*	Available content	Depth of Application's Knowledge
• *They have a lot of information and you can see kind of like during the night if it spikes when you woke up and it was pretty accurate that way and you could feel like a dream journal and put in you know if you had caffeine late and things like that to kind of track if that affects your sleep. (#7)*	Accuracy of data and content	
• *I'd probably explore other apps to see what's available beyond breathing and meditation for wellness apps I think. Anything that engages me more to either physically do something differently the breathing in meditation or mentally do something different. (#9)*<br>• *The training programs are always in depth and challenging. Because most apps start really easy and then go full board and you're unprepared. This is pretty hard, but it increases in a way that makes sense. (#3)*	Completeness	

Sample Responses	Initial Coding	Final Code
• *But there seem to be no consistent rules. It was overly complicated. I'm like I don't know how kids would play this. (#6)*<br>• *[I need] more transparency in what the app is offering. A clear direction or like knowing to maybe the app isn't for you. (#9)*<br>• *I don't know if it's because it's new but when I opened it up, there seemed to be multiple touch buttons on the screen that I wasn't sure. It looked like they were extra. So, I didn't know what they were there for. (#16)*<br>• *I don't know if it's a bug or if it's supposed to be that way but if you have to pause it doesn't work and it's like you didn't even do it. So, if you got through two of the three minutes it was as if you didn't even do it that day. Then the app locks you out for 17 hours or something like that and then you can't do another meditation. (#14)*	Process of the app<br><br>Clarity of rules and functions	System Rules
• *I guess to learn from it, learn from the app… So that it either gives me confirmation that I am doing well, or it will help me get better. (#1)*<br>• *Since I log food, it would be nice if it would look at what I eat and make some suggestions. (#7)*<br>• *[The app] tells me if I should increase the amount of activity …and it encourages me in a non-judgmental way … just a happy little voice thing. (#6)*<br>• *I noticed because when either friend was coming over or if I have a really busy day it would be difficult to commit the time for it to be effective, and so because I had to be using it every day that if I didn't log it again, it wouldn't give me a good average. (#2)*<br>• *I used it at the end of the day, almost exclusively. I think there were two or three times when I used it earlier in the day. Usually, it was at the end of the day and I would just enter the food, breakfast, lunch and dinner initially. (#4)*	Personalized progress analysis<br><br>Amount of usage time needed	Actionable Recommendations
• *I think Zombies Run would be effective for a lot of people. For me what they provide value in, like in their addons, does not work. I get [the value] in other places already. So, if I didn't do podcasts, that would be a really nice way to introduce you to walking and running (#6)*<br>• *I was constantly dehydrated… and I can't believe how much it [the app] helped because now I'm waking up in the middle of the night and I need my water. I have always read that you are supposed to really drink a glass of water, first thing when you wake up. I tried doing that on my own and never did [work]…. But the app me do it…if I do use it for a couple more weeks that will just become second nature to me (#13)*	Match between features and user needs	Fit Between User and System

Sample Responses	Initial Coding	Final Code
• *I feel like having only one means of communication or accountability is not good for me. I think if I'm serious about it, then I need to go to the meetings and be more engaged. Even though the system holds me accountable for it, it was not enough. (#8)*<br>• *I liked the idea of keeping online track of reps and weight that I take. But the whole app experience and having to put in all this idea did not work for me. (#5)*	Abandon use	Decision about use
• *It [my use] depends on whether I find the app useful or not, because meditation is something that seems really helpful for me and I'd like the idea, but the [app] implementation isn't working so well…so, I'll go try something else and see. (#10)*<br>• *I probably will continue… but now I'm out and I can't use the free content any more than I might sort of jump to another couple of apps that have the same kind of purpose. (#14)*	Switch application	
• *I think if I went on vacations, I would think to use it to see how much I am eating. Overall, I would occasionally use it but not often. (#2)*<br>• *Well I might use it periodically just to check myself, am I still close to my goal, or maintaining my goal. But I cannot see myself indulge in this much. Probably once every 3 months! (#4)*	Limit use	
• *Plant nanny I'm definitely going to continue using. I think you know the challenge is a group with 10 or 15 people strangers and I'm competitive, so I want to win. Some of the numbers were so low. So yeah, I would get second or third place. (#13)*<br>• *I'm going to keep it in my phone. I want to keep using it. So, trying to figure out what it costs but that should be okay. (#1)*	Continue use	
